# Refractory Escherichia Coli Pneumonia: A Case Report

**DOI:** 10.7759/cureus.35226

**Published:** 2023-02-20

**Authors:** Seyed A Khalafi, Alan De La Rosa Vaquez, Fatma Dihowm

**Affiliations:** 1 Paul L. Foster School of Medicine, Texas Tech University Health Sciences Center El Paso, El Paso, USA; 2 Internal Medicine, Texas Tech University Health Sciences Center El Paso, El Paso, USA

**Keywords:** e. coli pneumonia, refractory pneumonia, e. coli bacteremia, cavitary lung lesions, rapid deterioration

## Abstract

*Escherichia (E.) coli* pneumonia is a rare infection commonly presenting with a cavitary lesion. We report a case of a 44-year-old Hispanic male with comorbidities who was admitted to our facility with multiple falls for two days, shortness of breath, continuous diarrhea, and urinary urgency. Lab results showed leukocytosis with neutrophil predominance, anemia, and respiratory alkalosis. The patient was also noted to have uncontrolled diabetes mellitus with an A1c of 17.6%. Prior to admission to the medical intensive care unit (MICU), the patient was administered vancomycin and cefepime. The patient was then started on fluconazole while admitted to the MICU. In addition, a chest X-ray was conducted, showing patchy airspace opacities in the right upper lobe. A chest and abdominal CT also showed multiple cavitary lesions, pulmonary nodules, and nodular liver contour. Bronchoscopy with bronchoalveolar lavage conferred trimethoprim/sulfamethoxazole-resistant *E. coli* without fungal or acid-fast bacilli growth and was subsequently started on ampicillin/sulbactam. Infectious disease was consulted and advised to begin ertapenem. The patient developed increased respiratory demands and was subsequently started on mechanical ventilation with vasopressors. The patient was successfully weaned off and downgraded to the telemetry floor. The patient was successfully discharged in stable condition. This case highlights a severe and uncommon complication of *E. coli* infection causing pneumonia with cavitary lesions.

## Introduction

*Escherichia coli* (*E. coli*) pneumonia is considered a rare phenomenon. Most community-acquired pneumonia is caused by gram-positive bacteria, such as *Streptococcus pneumoniae* and *Staphylococcus aureus*, however, gram-negative bacteria are being recognized more often recently [1]. Previous studies have reported that *E. coli* make up only 3-12% of all community-acquired pneumonia pathogens [2-4]. It is believed that *E. coli* pneumonia may occur due to bacteremia or ventilator association [5]. Recently, the incidence of *E. coli* mechanical ventilation-associated pneumonia is more than *Pseudomonas aeruginosa* and *Staphylococcus aureus *[6-8]. There is very limited published literature on *E. coli* cavitary pneumonia, with most cases showing patients presenting in the intensive care unit (ICU) [9]. In this article, we report a case of refractory *E.coli* cavitary pneumonia diagnosed in a 44-year-old male with multiple comorbidities.

## Case presentation

A 44-year-old Hispanic male, with a past medical history of uncontrolled diabetes mellitus, hypertension, hypothyroidism, smoking history of more than 10 cigarettes per day for 24 years, pancolitis, and gout, arrived at the emergency room with a chief complaint of profound weakness with falls for two days, shortness of breath, continuous diarrhea, and urinary urgency. The patient was afebrile with a temperature of 36.9 degrees Celsius, had tachycardia (114 beats/min), hypotension (70/49 mmHg), non-tachypneic (18 breaths/minute), and had an oxygen saturation (SatO2) of 90% on room air. On physical exam, the patient was alert and oriented, but cachectic. The patient’s neck was supple and non-tender, with no jugular venous distention or lymphadenopathy. Lungs showed mild inspiratory rhonchi on the lower left lung with clear auscultation on the right and non-labored respiration. The patient had an elevated heart rate with regular rhythm and with no murmurs appreciated. The abdomen was soft, non-tender, and non-distended. The skin was also warm, dry, and pink with no obvious rashes or lesions.

Admission lab results showed a high white blood cell count with a high absolute neutrophil count and low hemoglobin and hematocrit. Additionally, low sodium and chloride were also noted on admission. Hemoglobin A1c and serum lactic acid were measured as high. Inflammatory markers showed a high erythrocyte sedimentation rate and a high C-reactive protein level. Arterial blood gas revealed a high pH, low partial pressure of carbon dioxide (pCO2), and low partial pressure of oxygen (pO2). The patient was also noted to have high thyroid-stimulating hormone and low serum T4 levels (Table 1).

**Table 1:** Summary of laboratory studies (total of 32 hospitalization days) HD: hospital day, WBC: white blood cells, HGB: hemoglobin, HCT: hematocrit, pCO2: Partial pressure of carbon dioxide, pO2: partial pressure of oxygen, *: Patient intubated

	HD 1	HD 6	HD 11*	HD 17	HD 27	Reference Ranges
pH	7.485		7.294	7.524		7.35 – 7.45
pCO_2_ (mmHg)	47.9		50.8	33.0		35 – 45
pO_2_ (mmHg)	27.5		63.3	87.8		80 – 100
WBC (x 10^3^/UL)	20.2	16.2	23.9	10.7	14.6	4.5 – 11.0
HGB (g/dL)	8.5	8.1	8.6	7.5	8.6	12.0 – 16.0
HCT (%)	24.7	25.1	26.6	23.1	26.8	38.0 – 47.0
Serum Sodium (mmol/L)	117	125	130	130	126	135 – 145
Serum Potassium (mmol/L)	4.4	4.1	5.3	4.4	5.1	3.5 – 5.1
Serum Chloride (mmol/L)	88	99	103	102	93	98 – 107
Serum Glucose (mg/dL)	422	124	86	159	140	74 – 106
Serum Lactic Acid (mmol/L)	2.6					0.7 – 2.1
Hemoglobin A1c (%)	>17.6					<5.7
Free Serum T_4_ (ng/dL)				0.35		0.78 – 2.19
Thyroid Stimulating Hormone (MIU/L)		56.9		78.8		0.465 – 4.680
Serum Cortisol (mcg/dL)					1.75	4.46 – 22.7

Due to concerns of bacteremia, a sepsis workup was conducted. Urinalysis showed yellow, turbid urine with moderate blood and high leukocyte esterase. Urine microscopy also revealed >50 WBC/hpf, >20 red blood cells/hpf, and occasional hyaline casts. Urine culture was positive for 50-100,000 colonies/mL with multiple species present due to possible contamination. The patient also had purulent penile discharge with cultures growing yeast and gram-positive rods and was also started on fluconazole. The patient was initially started on vancomycin and cefepime. Blood cultures were positive for *E. coli *resistant to trimethoprim/sulfamethoxazole (TMP/SMX), and sputum cultures had no growth for acid-fast bacilli but grew *E. coli *with similar resistance. The patient was subsequently started on ampicillin/sulbactam in accordance with sensitivities.

**Figure 1:**
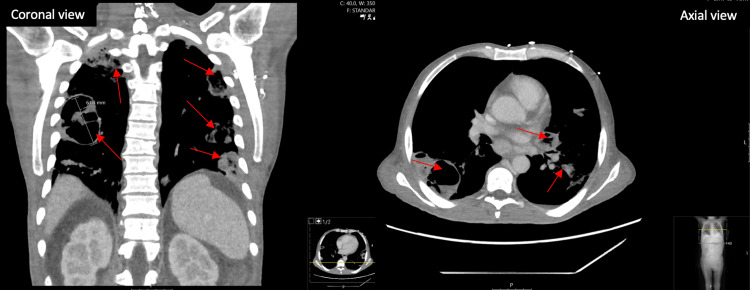
Initial chest CT with contrast in the coronal and axial views showing patchy airspace opacities in the upper lobe and interval development of multiple cavitary lesions Red arrows indicate cavitary lesions.

Initial chest X-ray and computed tomography (CT) with contrast on admission showed patchy airspace opacities in the upper lobe and interval development of multiple cavitary lesions and pulmonary nodules with reactive mediastinal lymph nodes and anasarca, respectively (Figure 1). In addition, an initial abdominal CT was performed that revealed a nodular liver contour that was concerning for underlying chronic liver disease and third-spacing with severe mesenteric edema and anasarca.

**Figure 2:**
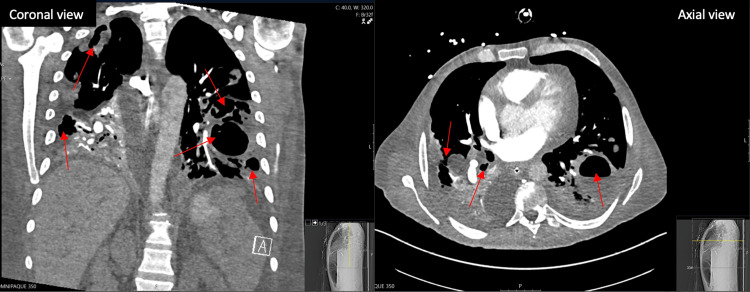
Chest CT angiography with contrast in the coronal and axial views 10 days after admission showing no signs of pulmonary embolism and worsening cavitary lesions Red arrows indicate cavitary lesions.

**Figure 3:**
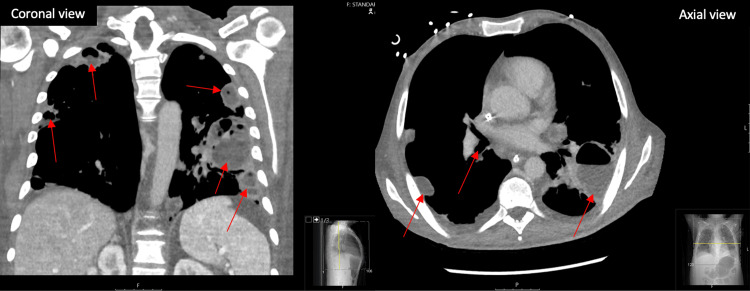
Chest CT with contrast in the coronal and axial view 14 days after admission showing worsening signs of cavitary lesions Red arrows indicate cavitary lesions.

The patient was then admitted to the medical intensive care unit (MICU) due to hemodynamic instability. While in the MICU, the patient had worsening leukocytosis with neutrophilic predominance. A bronchoscopy was subsequently performed with bronchoalveolar lavage (BAL) analyzing for fungal, acid-fast bacilli, and bacterial, which were negative for all results except *E. coli *with trimethoprim/sulfamethoxazole (TMP/SMX) resistance. The patient was also seen in the hospital five months prior for pneumonia and had blood and BAL cultures that grew *E. coli* with similar resistance. Once stabilized, the patient was then transferred to the floor five days later for evaluation. A repeat chest X-ray three days later showed worsening bilateral severe airspace disease and consolidations with cavitary lesions. Infectious disease was consulted and recommended the patient be placed on ertapenem. On the ninth day of admission, the patient began to desaturate at 82% on a 4-liter high-flow nasal cannula and was intubated on mechanical ventilation with vasopressor support requiring repeat MICU admission. CT angiography showed no signs of pulmonary embolism but did show worsening cavitations (Figure 2). Repeat chest CT with contrast four days later showed worsening cavitated changes in the left lower lobe when compared to previous imaging (Figure 3). Interventional radiology was consulted for possible cavitary lesion biopsy but was deferred due to the risk of pneumothorax and the patient’s current condition.

After nine days on mechanical ventilation, the patient was weaned off successfully and transferred to the floor after stabilization. During floor transfer, the patient was afebrile with a temperature of 36.2 degrees Celsius, non-tachycardic (99 beats/min), normotensive (93/61 mmHg), tachypnea (18 breaths/minute), and had a SatO2 of 93% on room air. Repeat blood cultures on the floor showed no growth. While on the floor, the patient was also noted to have adrenal insufficiency with improvement on steroids, recommended by endocrinology consultation, and was discharged in stable condition.

## Discussion

The finding of cavitary lesions on chest CT in our case may be suggestive of infection, septic emboli, systemic inflammatory disease, or cancer. With a positive BAL culture and blood culture growing *E. coli*, we suspect the lesions to be due to *E. coli* infection. With this test confirming *E. coli* pneumonia prior to mechanical ventilation, our patient may have had community-acquired pneumonia or *E. coli *bacteremia possibly due to UTI, but inconclusive due to a contaminated urine sample. Our patient was also noted to have rapid deterioration, emphasizing the urgent need for early identification. During discharge, the patient was also noted to have adrenal insufficiency, which was not observed during early hospitalization, suggesting the patient developed this condition due to the infection. Patients who are susceptible to *E. coli *pneumonia include those who have comorbid conditions such as immunosuppression (31.7%), diabetes mellitus (18.5%), chronic alcohol consumption (23%), chronic respiratory disease (13.6%), and chronic heart failure (17.7%). It was also noted that 57% of the *E. coli *isolates in patients with pneumonia were of urinary origin [5]. Of all the types of pathogenicity of *E. coli*, pneumonia is the least discussed in many studies [1,10].

Our case supports the increased incidence of *E. coli *pneumonia in critically ill patients. A study conducted by La Combe et al. resulted in 260 *E. coli* isolates from 243 ICU patients who were predominantly male. One hundred seventeen were ventilator-associated, 61 were hospital-acquired, and 82 were community-acquired [5]. Another study conducted by Okimoto et al. presented with 22 patients admitted to the ICU with *E. coli* hospital-acquired pneumonia who were also predominantly male. Additionally, seven patients had underlying UTI infections while admitted [11]. Patients with *E. coli* pneumonia were more commonly to present with bacteremia. *E. coli* that are associated with bacteremia and sepsis were associated with virulence factors alpha-hemolysin and cytotoxic necrotizing factor type-1, which were also commonly found in uropathogenic *E. coli* [12]. As for our case, the patient was a male also diagnosed with multiple comorbidities, including diabetes mellitus, hypothyroidism, and adrenal insufficiency, and having a possible underlying UTI infection, suggesting an increased risk of *E. coli *pneumonia from bacteremia spread in critically ill patients. Males may also be more susceptible to more severe pneumonia symptoms due to hormonal influences [13]. Furthermore, the age range reported in the studies was 52-73 years old while our patient at age 44 years old was below the age range [5].

Patients who have contracted E. coli pneumonia also present with rapid deterioration. The study by John et al. presented 1029 patients with *E. coli *pneumonia, of which 40% required ICU admission, 20% required respiratory support, and 14% in-hospital mortality rate. These patients also had a higher mortality rate compared to gram-positive associated community-acquired pneumonia, however, had similar rates compared to other gram-negative bacteria [1]. In addition, the pneumonia PORT study recorded patients with *E. coli* pneumonia as having a pneumonia severity index of 4-5, representing expected mortality as high as 27% at 30 days and 21% at 90 days [14]. In the present case, we present a patient that also had rapid decompensation that required MICU admission twice and mechanical ventilation.

## Conclusions

In this case report, we present a rare type of pneumonia that presents with cavitary lesions. Our patient had multiple comorbidities, such as uncontrolled diabetes mellitus, hypothyroidism, and pancolitis, which may have increased his predisposition to this type of infection. Our case also portrayed a multidisciplinary approach, with infectious disease, interventional radiology, pulmonology, and endocrinology all included in the patient’s care. With *E. coli *being a common cause of infection, we hope to promote awareness of considering *E. coli *as a potential cause of pneumonia with cavitary formation.
